# Electronic health records: a new tool to combat chronic kidney disease? 

**DOI:** 10.5414/CN107757

**Published:** 2013-01-15

**Authors:** Sankar D. Navaneethan, Stacey E. Jolly, John Sharp, Anil Jain, Jesse D. Schold, Martin J. Schreiber, Joseph V. Nally

**Affiliations:** 1 Department of Nephrology and Hypertension, Glickman Urological and Kidney Institute,; 2 Cleveland Clinic Lerner College of Medicine of CWRU,; 3 Department of Internal Medicine, Medicine Institute,; 4 Department of Quantitative Health Sciences, Cleveland Clinic, and; 5 Explorys Corporation, Cleveland, OH, USA

**Keywords:** chronic kidney disease, electronic health records, research, quality of care

## Abstract

Electronic health records (EHRs) were first developed in the 1960s as clinical information systems for document storage and retrieval. Adoption of EHRs has increased in the developed world and is increasing in developing countries. Studies have shown that quality of patient care is improved among health centers with EHRs. In this article, we review the structure and function of EHRs along with an examination of its potential application in CKD care and research. Well-designed patient registries using EHRs data allow for improved aggregation of patient data for quality improvement and to facilitate clinical research. Preliminary data from the United States and other countries have demonstrated that CKD care might improve with use of EHRs-based programs. We recently developed a CKD registry derived from EHRs data at our institution and complimented the registry with other patient details from the United States Renal Data System and the Social Security Death Index. This registry allows us to conduct a EHRs-based clinical trial that examines whether empowering patients with a personal health record or patient navigators improves CKD care, along with identifying participants for other clinical trials and conducting health services research. EHRs use have shown promising results in some settings, but not in others, perhaps attributed to the differences in EHRs adoption rates and varying functionality. Thus, future studies should explore the optimal methods of using EHRs to improve CKD care and research at the individual patient level, health system and population levels.

## Introduction 

Chronic kidney disease (CKD) is a public health problem across the globe in both developed and developing countries [1, 2, 3, 4]. In the United States, Medicare spends over 33 billion dollars annually to provide care for CKD patients [5]. CKD is a silent disease in its early stages and there is low awareness of its presence both among those who are affected and those who are treating them [6, 7]. Various interventions such as early referral to nephrologists have been developed to address the challenges of early identification and to improve the quality of care among patients identified with CKD, but these have had only limited success [8]. Thus, the search for novel tools to continue to recognize CKD at an earlier stage and address the barriers in CKD care must continue. 

In the United States, the Institute of Medicine has called for increasing the use of electronic health records (EHRs) since the 1990s. However, the adoption rate of EHRs has been low leading to the passage of the Health Information Technology for Economic and Clinical Health (HITECH) Act in 2009; now, adoption in the United States has significantly increased the number of medical practices who use EHRs [9]. Furthermore, the use of EHRs is increasing in both developing and developed countries [9, 10, 11, 12, 13, 14] leading to widespread use of EHRs by nephrologists. In this article, we will review the structure and function of EHRs, its role in early identification and management of CKD patients, and its potential uses in clinical research related to kidney disease. 

## Structure and function of EHRs 

When they began in the 1960’s, EHRs were simply computerized systems that allowed for document storage and retrieval. With the advancement of technology and more widespread adoption, they have evolved into a much more sophisticated tool. By definition, the essential components of an EHR include reporting of data relating to patient health information, electronic communications between providers, patient support and administrative support [15]. Reporting of data can be further delineated into areas that support additional use of health data at the individual provider, clinic, or health system level in the form of quality reports, clinical research, and public health [16]. The ability of EHRs to store and retrieve structured data longitudinally such as demographics (age, gender and ethnicity), laboratory results, clinical data such as blood pressure or heart rate, standardized medications and standardized diagnoses is instrumental in datasets required for meaningful clinical research [17]. Standardized data from EHRs can be used in clinical research to generate a sample for a case control study, construct a cohort, or identify people with certain conditions or outcomes [18]. 

### Role of EHRs in patient care and research 

Studies have shown that among health centers with EHRs, there was an increased adherence to guideline-based care, enhanced surveillance and monitoring, and decreased medication errors [19]. Physicians who use EHRs believe that they improve the quality of patient care [20] and that the data is superior to claims or administrative data for quality reporting [21]. A study from Better Health Greater Cleveland, a Robert Wood Johnson Aligning Forces quality project in the United States showed that across all insurance types, use of EHRs were associated with significantly higher achievement and greater improvement in diabetes quality of care than those centers with paper records in the study [22]. In addition, among elderly patients, electronic medical record reminders alone facilitated improvement in vaccination rates [23]. Also, a recent Cochrane review demonstrated the effectiveness of EHRs-facilitated interventions on smoking cessation support actions by clinicians [24]. Yet, while processes of care have been shown to improve with EHRs and automated reminders or decision support systems, quality of care is not always improved and clinical outcomes may not change [25, 26, 27]. 

Building on the potential of EHRs in clinical research, the National Human Genome Research Institute formed a consortium to develop, disseminate and to use methods that combine DNA biorepositories with EHRs systems for large-scale, high-throughput genetic research [28]. In addition, we have observed significant benefits of EHRs in primary care settings. For example, in the setting of fully implemented EHRs with a disease registry, cholesterol management of patients with diabetes was significantly improved [29]. Moreover, public health alerts in EHRs systems can be useful in reporting, recommending specific tests, as well as suggesting secondary prevention [30]. Lastly, well designed patient registries allow for improved aggregation of patient data for quality improvement to the facilitation of clinical research [31, 32, 33, 34, 35]. 

## EHRs in CKD management 

### Early identification of CKD

Effective utilization of EHRs could help in improving both the identification of CKD patients and the quality of care delivered to them. Previous studies have documented how the under-diagnosis of CKD by primary care physicians leads to late referral to nephrologists for CKD care and its untoward consequences [7, 36]. Automated clinical alerts using EHRs may help to diagnose CKD earlier and improve referral rates. Recent reports from Canada, Australia and other developed countries have shown promising data that automated estimated glomerular filtration rate (eGFR) reporting in laboratory results improve nephrology referrals [37, 38]. It remains to be seen yet whether these referrals improve patient-centered outcomes; however, this could be the first step towards better results. A major limitation of automated eGFR reporting is that it is not specifically patient-centered and may trigger unnecessary referrals. In contrast, Clinical Decision Support Systems in EHRs that combine programmed expert rules or clinical practice guidelines and individual patient data can provide patient-specific recommendations to the physicians and other care providers. A pilot single-center clustered randomized clinical trial examined whether Clinical Decision Support Systems improve CKD care through increased nephrology referrals. Results of this trial suggested the feasibility of examining Clinical Decision Support Systems in future clinical trials to improve CKD care in outpatient setting [39]. 

### Management 

Management of CKD requires coordination of care between physicians from various specialties and allied health care workers that can be daunting. Effective utilization of EHRs could help in developing programs that facilitate efficient exchange of information between caregivers. Recently, Kaiser Permanente of Hawaii developed a proactive nephrology referral using their electronic database within their health care system [40]. During this pilot phase, primary care physicians of patients who were at high risk for disease progression (based on EHRs documentation and risk stratification) were solicited to refer patients to nephrologists for CKD care. Subsequently, with the help of EHRs and based on the support of physicians seen in the pilot phase, unsolicited consultations were provided to the primary care physicians by nephrologists for high risk CKD patients. This unconventional referral system using EHRs improved AVF placement prior to starting dialysis and more patients started dialysis as outpatients rather than in an inpatient setting. 

Veterans Health Affairs (VHA), one of the largest integrated health care systems in the United States, cares for over 5 million patients using system-wide EHRs for both administration and clinical care since 1985. In early 2000, VHA developed and implemented tools for primary care providers to manage CKD patients including making appropriate referrals to prepare patients for renal replacement therapy in the future. Apart from improving the management of comorbid conditions in CKD patients, this has resulted in an increasing number of patients starting dialysis using an arteriovenous fistula rather than initiating dialysis using a dialysis catheter among VHA patients [41]. 

## Cleveland Clinic experience 

### EHRs 

The Cleveland Clinic health system is an integrated delivery network comprised of the Cleveland Clinic main hospital, its 15 family health centers and 8 community hospitals in Northeast Ohio, USA. We serve an estimated population of over 1.5 million people with 75% of patients coming from the 7 counties adjacent to Cleveland and the rest from other areas. Our health care system uses an integrated ambulatory and inpatient EHRs system (Epic, Epic Corp., Verona, WI, USA) with a common patient index at all facilities since 2002. EHRs use is mandated for scheduling, order entry, documentation of progress notes, results review, medication management and provider-to-provider and provider-patient communication in all ambulatory sites affiliated with our health care system. Cleveland Clinic eResearch, a division within our health care system extracts relevant clinical data from the EHR and helps clinical researchers develop databases for use in research studies. 

### Development of a EHRs-based CKD registry 

We developed a EHRs-based CKD registry at the Cleveland Clinic with the intent of identifying CKD patients earlier and systematically in order to develop programs for CKD patients, well poised for outcomes research and create intervention programs to improve CKD care. As a first step, patients who had at least 1 face-to-face outpatient encounter with a Cleveland Clinic health care provider and a) had 2 eGFR values < 60 ml/min/1.73 m^2^ more than 90 days apart as of January 1, 2005 and/or b) were designated with International Classification of Diseases (ICD-9) codes for kidney disease (used twice in an outpatient encounter) were included in this registry. Our registry is comprised of 65,116 patients (57,440 patients with 2 eGFR < 60 ml/min/1.73 m^2^ and an additional 7,676 patients who met the ICD-9 code criteria) as of November 2011. Our CKD Registry was originally developed utilizing eGFR calculations that employ the Modification of Diet in Renal Disease (MDRD) formula, but we have also examined the implications of the new Chronic Kidney Disease Epidemiology Collaboration (CKD-EPI) equation upon patient characteristics and comorbid disease [42]. We are identifying patients who had proteinuria or albuminuria assessments within our health care system to identify additional high risk CKD population. The kappa statistics to assess the extent of agreement between the administrative dataset derived from the EHRs and actual EHRs chart review overall was excellent for different kidney diseases such as glomerulonephritis (λ statistic 0.85), polycystic kidney disease (λ statistic 0.90) etc. suggesting the reliability of the registry for outcomes research. The development and validation of our EHRs-based CKD registry have been described in detail elsewhere [43]. 

### Health services research 

Administrative data or claims data are available from federal agencies, health departments, insurance providers and other governmental agencies. These data provides comprehensive details about whether patients had physician visits, obtained relevant laboratory testing or imaging studies and had screening procedures thereby providing detailed cost incurred in health care delivery [44, 45]. However, often they lack extensive clinical details and may underestimate the presence of comorbid conditions. In contrast, EHRs provide much more reliable longitudinal clinical data than administrative data sets which is important for the study of disease progression, prognosis and outcomes [16]. This is particularly relevant for studying chronic disease conditions like CKD. 

**Figure 1.**
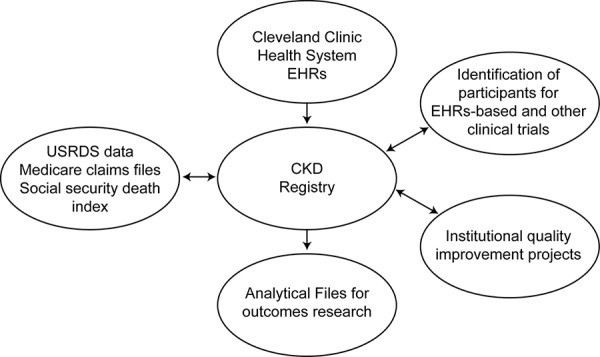
CKD registry and its role in research and patient care.

In an open health care system (where patients might get cared for not only in our health care system but also in other hospitals or clinics), EHR data of one institution alone may not be sufficient and need to be supplemented from other resources. As such, we have merged our registry participants with United States Renal Data System and Social Security Death Index to identify patients who have progressed to ESRD and who had expired (Figure 1). This has enabled us to conduct important outcomes research projects related to CKD complications and outcomes such as mortality while understanding the limitation that such projects are restricted to a single health care system [46, 47, 48]. In Northeast Ohio, efforts are being made to facilitate health information exchange across various institutions that may not only facilitate the data transfer in EHRs for clinical care but may provide additional data for research purposes. Lastly, care for non-dialysis dependent CKD is associated with higher costs (compared to non-CKD population) and such registries could also provide an opportunity to examine costs incurred for various management aspects of CKD [45, 49]. 

### Personal health record 

**Figure 2.**
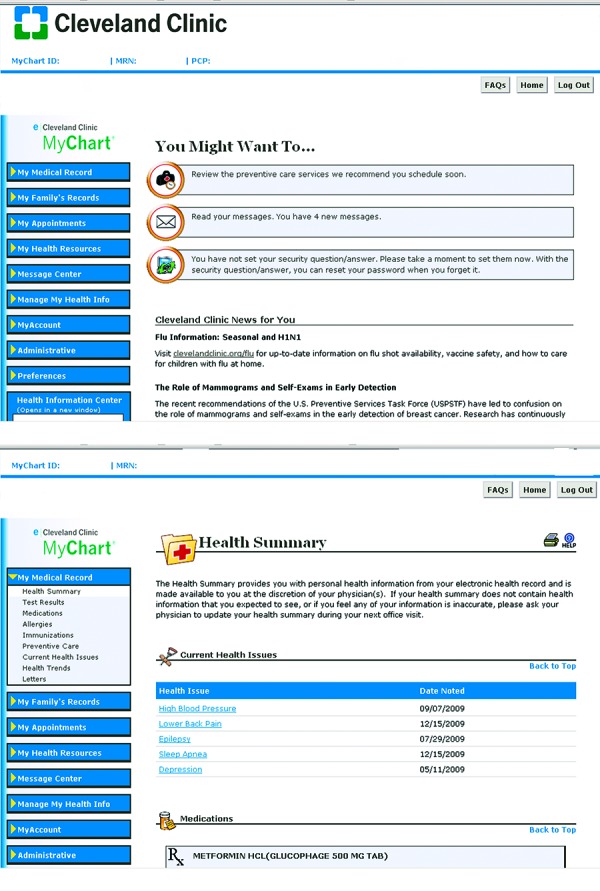
Overview of Personal Health Record (Panel A) and health details available in our Personal Health Record (Panel B).

In addition to the provider-based EHRs, Cleveland Clinic health system offers all patients the ability to access their medical information via a personal health record (PHR) tethered to their Cleveland Clinic health system EHRs (Epic MyChart, Epic Corp., Verona, WI, USA). A PHR is generally defined as an electronic application through which individuals can access, manage and share their health information with their health care providers. A spectrum of PHR is available, from stand-alone systems to those that are web-based and interface with the individual health system EHRs. Our system allows patients to activate their PHR accounts either in person or via an online authorization process. Once activated, patients can navigate around this secure web-based application and manage their health information. They can review and schedule appointments, request prescription renewals, view health summaries, access a current list of medications and review test results (Figure 2). Moreover, patients also receive automated important health reminders based on gender- and age-based health maintenance schedules, as well as chronic disease-related reminders (e.g., diabetes). Links within the PHR allow patients to access reliable health information about a broad range of topics of personal interest through a third-party vendor such as MedlinePlus. Secure messaging between the patient and provider is also available via the PHR to facilitate communication. 

### Ongoing EHRs based clinical trial 

We are examining the effects of innovative interventions for CKD on the decline in renal function among patients with CKD Stages 3b or 4 (eGFR 15 – 45 ml/min/1.73 m^2^). We are developing a CKD Patient Navigator program, adapting from the use of Patient Navigators successfully in the field of oncology [50]. In addition, an enhanced PHR that will use electronic communication to disseminate CKD stage-specific goals of care and CKD education to patients is being developed. These features help us conduct a randomized controlled trial using a factorial design to investigate the clinical impact of the two interventions, a CKD Patient Navigator and enhanced PHR, compared to usual care for CKD Stage 3b/4 patients. Primary outcome measures of this study include various processes of care for CKD patients including referrals for dialysis access and renal transplantation. With the help of our CKD registry, we are recruiting patients for this clinical trial from outpatient clinics at main campus and family health centers affiliated with our health care system. 

### Recruitment for other clinical trials 

EHRs can be used to identify potential study participants to be recruited into the clinical trials [51]. Thus far, we have been using EHRs to identify eligible participants for ongoing clinical trials in CKD in our center such as the Systolic Blood Pressure Intervention Trials (SPRINT). In addition, data relating to study participants that may be collected in clinical care but not in a formal study setting may be obtained for ancillary study purposes. 

## CKD registries and surveillance projects 

Different models have been adopted to identify CKD patients and obtain pertinent clinical details relevant to them. The Alberta Kidney Disease Network in Canada has identified CKD patients through laboratory data and has further enriched this database with other health record files from their province [52]. Likewise, Kaiser Permanente (a US based insurance company) has developed a CKD registry similar to that of the Cleveland Clinic [53]. These efforts have facilitated the achievement of high-quality outcomes research projects and helped to conduct clinical trials in CKD along with implementation of projects to improve the quality of care delivered to CKD patients. In the United States, the Center for Disease Control and prevention has initiated a CKD surveillance program to identify and collect data relating to CKD patients from various institutions/health care systems across the country [54]. These include both CKD patients identified both through EHRs at various institutions (such as the VHA system), and also from prospective cohort studies, clinical trials, and other administrative data sets. 

## Limitations of EHRs 

A major limitation of EHRs is the human and capital resources associated with its implementation rendering inequalities among health care settings in adopting the EHRs. However, with the incentives offered by the federal government, implementation and use of EHRs has increased across the United States. This might not be the case in several developing and under-developed countries (that represent a significant proportion of CKD population) thereby limiting the use of EHRs. Privacy rules relating to protecting personal health information must be adhered to while seeking access to EHRs data to perform research. Therefore, it is customary to use de-identified data in the United States where unique national patient identifier is lacking in contrast to other countries such as Australia, Canada and European Union that have unique patient identifiers [55, 56]. EHRs and health information exchange is in its infancy in several countries including the United States which may limit its practical applications and proposed benefits [57]. One particular issue in CKD is that even in institutions with well-developed EHR, data relating to death and dialysis might not be readily available. Also, the usefulness of the PHR relies on the level of engagement of patients with their health rather more so than just access to and the use of PHR itself [58]. Thus, adoption of PHR should involve patient education akin to provider education that is needed to improve EHRs use. 

## Conclusions 

Identifying novel methods to develop programs that improve patient care and obtaining high-quality data for research projects in Nephrology has remained a challenge. Available data and our/others experience with EHRs has shown promising results to address some of these challenges. With the higher use of EHRs in developed countries and ongoing efforts in other developing countries to promote EHRs, the influence of EHRs will rise in future. EHRs-based interventions have show mixed results in the literature in other specialties warranting careful application of this emerging technology for clinical care and research among CKD patients. 

## Disclosure 

SDN is supported by a career development award from the National Center for Research Resources and the National Center for Advancing Translational Sciences, National Institutes of Health (Grant #RR024990). SEJ is supported by the National Institutes of Health career development award 1K23DK091363 and DK094112. JVN is supported by DK094112. JDS is supported by NIH/NIDDK (R01 DK085185 and DK094112) and investigator initiated-grant support from PhRMA foundation, Genzyme and Roche Organ Transplant Research Foundation. The contents of this manuscript is solely the responsibility of the authors and does not necessarily represent the official views of the NIH. The authors have no relevant financial interest in the study. The creation of the CKD registry was funded by an unrestricted grant from Amgen, Inc., Thousand Oaks, CA, USA, to the Department of Nephrology and Hypertension Research and Education Fund. 

## Acknowledgments 

We thank other members of the Cleveland Clinic CKD registry (Susana Arrigain, Vicky Konig, Welf Saupe, Travis Moore, Jennifer Hyland, CNP and James Simon, MD) for their contributions to the CKD registry development and maintenance.
